# Experiences of piloting integrated bite case management in Zaria Metropolis, Nigeria

**DOI:** 10.3389/fitd.2026.1757621

**Published:** 2026-02-13

**Authors:** Grace Sabo Nok Kia, Mathias Samuel, Elaine A. Ferguson, Ishaq Ibrahim, Kennedy Lushasi, Jerome Okpanachi, Nai Rui Chng, Jacob Kwada Paghi Kwaga, Katie Hampson

**Affiliations:** 1 Department of Veterinary Public Health and Preventive Medicine, Faculty of Veterinary Medicine, Ahmadu Bello University Zaria, Zaria, Kaduna State, Nigeria; 2 African Centre of Excellence in Neglected Tropical Diseases and Forensic Biotechnology, Ahmadu Bello University Zaria, Zaria, Kaduna State, Nigeria; 3 War Against Rabies Foundation, Kaduna, Kaduna State, Nigeria; 4 Department of Livestock Services, Kaduna State Ministry of Agriculture and Rural Services, Zaria, Kaduna State, Nigeria; 5 School of Biodiversity, One Health & Veterinary Medicine, University of Glasgow, Glasgow, United Kingdom; 6 Environmental Health and Ecological Sciences Department, Ifakara Health Institute, Ifakara, Tanzania; 7 Saint Jerome Veterinary Services Zaria, Zaria, Kaduna State, Nigeria

**Keywords:** animal investigation, IBCM, lyssavirus, One Health, post-exposure prophylaxis, risk assessment, surveillance, Zero by 30

## Abstract

**Background:**

Rabies remains a major public health risk in Africa and is estimated to cause over 1,600 deaths annually in Nigeria. Integrated Bite Case Management (IBCM) is recommended as a One Health approach for rabies surveillance but has yet to be implemented within Nigeria.

**Methods:**

Our aim was to gain epidemiological and operational understanding of implementing IBCM within Sabon Gari Local Government Area (LGA) of Zaria Metropolis, Kaduna State, Nigeria through an implementation research approach. We developed an IBCM protocol with local practitioners and piloted it from April 2023 until December 2024, and analyzed resulting data.

**Results:**

We identified very low access to rabies post-exposure prophylaxis (PEP) within the study area. Although incidence of bite patients was low (~0.98/100,000/year, 95% confidence intervals, CI: 0.68-1.42), a large proportion were identified as rabies exposures (41%) and two human rabies deaths occurred (0.11 deaths/100,000/year, 95%CI: 0.031-0.41), corresponding to very low healthcare seeking (0.50 probability of rabies exposures receiving PEP, 95%CI: 0.27-0.86). Investigations triggered by dog bite incidents or community notifications identified probable rabid dogs from clinical signs/history (45% of investigated dogs), with all recoverable samples (8) confirmed positive by rapid testing, including three dogs that died in quarantine. IBCM revealed the dog meat trade as a sentinel for rabies detection, with three rabid dogs sold for consumption. Key aspects of IBCM were refined to improve implementation during the pilot.

**Conclusions:**

Piloting IBCM revealed a low incidence of bite presentations reflecting the small dog population (high human: dog ratio) in these communities and very low levels of care seeking among those at risk. We conclude that there is an urgent need to simultaneously improve PEP access and raise awareness about the dangers of rabies. Other critical gaps in rabies control and prevention, include low dog vaccination coverage, limited training in rabies prevention and a lack of resources for surveillance, despite urgent demand and enthusiasm for implementation. The generalizability of our conclusions are limited given our experiences are derived from a single LGA. Nevertheless, this we provide lessons for how to develop IBCM for this local health and veterinary context in accordance with cultural norms and identify considerations for the design and implementation of IBCM elsewhere.

## 1 Introduction

Rabies remains a critical public health problem in Nigeria despite being 100% vaccine-preventable. Globally, more than 59,000 people die from rabies globally each year, mostly in Asia (59.6%) and Africa (36.4%) (1). The vast majority of these human rabies cases result from dog bites (2). Once the rabies virus enters a host it targets the brain and spinal cord and causes damage to the nervous system that results in clinical symptoms with no chance of recovery (3).

The control of rabies at source through the vaccination of domestic dogs is crucial to the prevention of human rabies (4, 5). After a potentially rabies dog bite, effective management involves timely post-exposure prophylaxis (PEP), comprising wound washing, a course of vaccination and, in severe exposures, rabies immunoglobulins infiltrated at the wound site, to prevent clinical signs manifesting (6, 7). The requirement to address the disease in dog populations, while also ensuring emergency preventative care for bite victims, means that rabies demands a One Health approach. One Health is a collaborative, multisectoral, and transdisciplinary approach that aims to sustainably balance and optimize the health of people, animals and ecosystems, recognizing the inter-dependent relationships between people, animals, plants, and their shared environment (8).

Global One Health governance is crucial in rabies elimination. The tripartite, comprising the Food and Agriculture Organization of the United Nations (FAO), World Health Organization (WHO), and World Organisation for Animal Health (WOAH), lead the ‘Zero by 30’ initiative to end dog-mediated human rabies by 2030 (9, 10). The ‘Zero-by-30’ strategic plan recommends operationalizing One Health principles for rabies surveillance (11, 12). Surveillance plays a crucial role in disease control and elimination (13, 14). It involves the ongoing, systematic collection, analysis, and interpretation of infectious disease data, guiding timely interventions (15, 16). However, in many low-income countries, surveillance for guiding rabies control remains inadequate. Practical measures are needed to enhance surveillance, specifically to improve timely case detection, so as to inform rabies control and prevention (10, 17–19).

Integrated bite case management (IBCM) is an intersectoral collaborative approach to rabies surveillance that engages health workers, veterinarians, and laboratory professionals to monitor and respond to potential rabies cases in the community (10). The use of IBCM has shown benefits across various countries, including the Philippines, Haiti, Sri Lanka, Tanzania, Chad, Bangladesh, Ethiopia, and Vietnam (10, 20–25). IBCM assesses the health status of animals involved in bites for informed decisions about provisioning PEP to exposed individuals and for removing suspected rabid animals from communities (11). By targeting PEP use, IBCM is intended to reduce human deaths by ensuring access to and availability of PEP for those at risk (14, 26). However, Nigeria faces significant challenges in rabies surveillance and has not yet adopted IBCM (27, 28).

Approximately 1,600 human rabies deaths occur annually in Nigeria, based on modeling estimates that account for underreporting and diagnostic limitations (1). Nigeria’s Integrated Disease Surveillance and Response (IDSR) strategy includes suspected human rabies deaths but covers less than 50% of the country’s health facilities. Challenges to the implementation of IDSR include insufficient reporting tools and trained personnel (18, 19, 28). The situation for animal health surveillance is worse and lacks the capacity for outbreak investigation and response. Following the 2006 outbreak of highly pathogenic avian influenza H5N1 in Nigeria, the National Animal Disease Information System (NADIS), a web-based and mobile-enabled application, was introduced to receive and conduct real-time disease reporting, analysis, and georeferencing of disease outbreaks (19, 27).

Subsequently, diseases such as rabies were incorporated into NADIS to gather surveillance data from veterinary hospitals and clinics across the country. However, despite training and provision of equipment, implementation is lacking. Moreover, there is poor linkage and data sharing needed to guide rabies control and prevention measures between the public health and veterinary sectors in Nigeria (17, 19). Although the Nigeria Centre for Disease Control (NCDC) has successfully implemented projects using the One Health approach and has formulated policy documents, including the One Health National Strategic Plan (28), the situation at the sub-national level presents a contrasting picture, where the operationalization and institutionalization of One Health remain suboptimal (17, 19).

Gavi, the Vaccine Alliance’s investment strategy to improve access to rabies PEP, also promotes the use of IBCM (29). Rabies-endemic countries can now apply to Gavi for human rabies vaccines, with the aim of catalysing action on rabies control (30, 31). To successfully implement IBCM, requires customization to local contexts given differences in health and veterinary systems, healthcare practices and cultures around dog ownership (11, 32). In Nigeria, variation in rabies surveillance across states and Local Government Areas (LGAs) necessitate tailored strategies. For example, Gombe State adapts and actively implements a collaborative network of speedy information transfer between veterinarians and medical professionals following a dog bite, which has been helping to enhance the state’s rabies surveillance and control (19). Nigeria’s human–dog relationship is shaped by diverse sociocultural factors, spanning security and hunting, urban pet-keeping and purebred ownership, Islamic norms that permit dogs for guarding, herding, or hunting while discouraging unnecessary indoor pet-keeping, and localized dog-meat trade (26, 33). Together, these cultural practices affect rabies management and prevention across the country.

Here, we investigate aspects of rabies management and the operationalization of the One Health approach in Sabon Gari LGA, Kaduna State in Northwest Nigeria. We focus on piloting IBCM to gain understanding on how to improve both public health outcomes and animal health surveillance. Despite research on rabies in Nigeria, this pilot provides the first implementation research on IBCM in the country, generating practical insights to inform scale-up, improve PEP use, and strengthen One Health collaboration across public health and veterinary services.

## 2 Materials and methods

We applied an implementation research design to pilot IBCM in Sabon Gari LGA of Kadua state. As this is the first example of IBCM that we know of in Nigeria, we document our experiences with the aim of gaining understanding of how IBCM can be effectively operationalized and to learn lessons to support further contextual tailoring and support for large-scale implementation.

### 2.1 Study area

**Figure 1:**
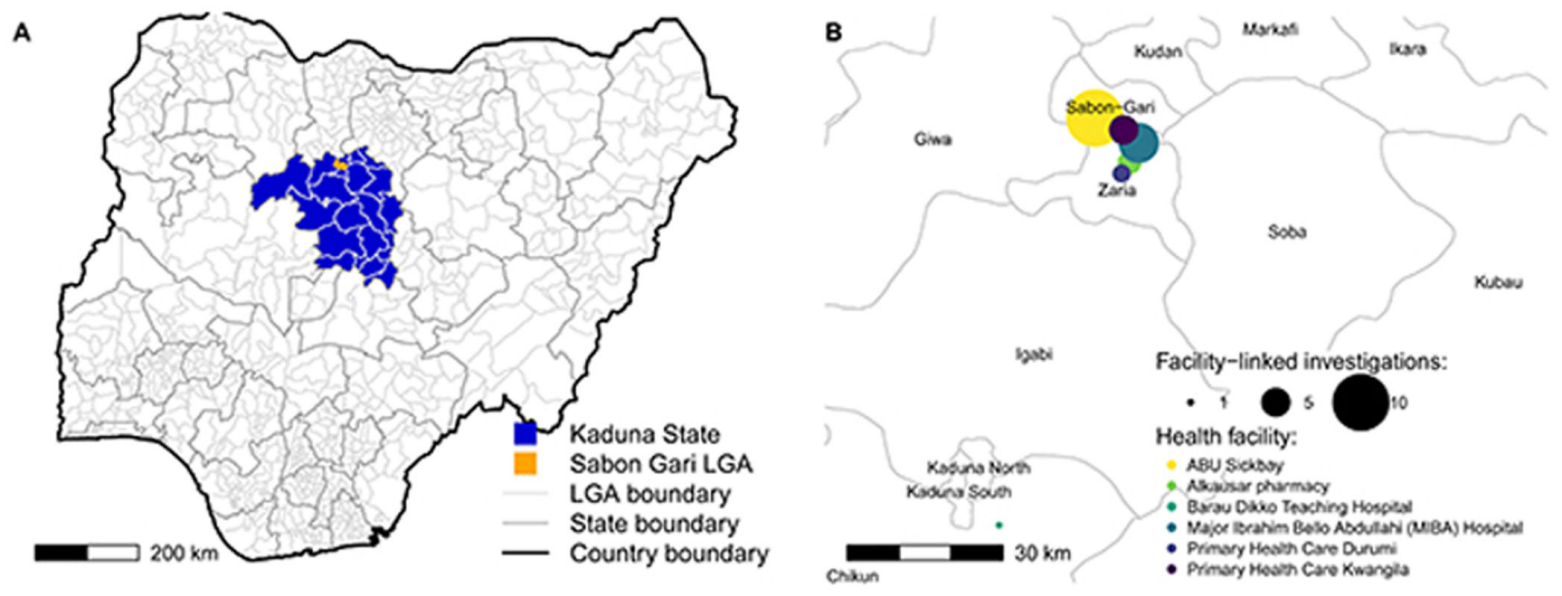
Study area within Nigeria for piloting IBCM. **(A)** Map of Nigeria indicating Sabon Gari LGA (orange), within Kaduna state (blue). **(B)** Health Facilities in Sabon Gari LGA that were enrolled to the IBCM pilot, and additional facilities added following bite patient referrals. Circle size indicates the number of animal investigations initiated from these facilities. Grey lines indicate LGAs.

The study was carried out in the Sabon Gari LGA of Kaduna State, situated in the Guinea Savannah zone of Northwest Nigeria (Figure 1A). The LGA covers an approximate land area of 600 km^2^ and has a human population of around 430,500 in 2022, according to projections from the 2006 national census using the population growth rate for Kaduna state (34). Sabon Gari is one of 23 LGAs within Kaduna State and 4 LGAs within the Zaria metropolis. Sabon Gari LGA comprises two districts: Sabon Gari and Basawa, which collectively encompass 11 wards (34).

The Sabon Gari LGA has 22 primary health care (PHC) facilities, 25 registered private hospitals, and 12 institutional clinics associated with training institutions (35). The Ahmadu Bello University (ABU) Sickbay also referred to as University Health Services is located less than 8 km from the center of Sabon Gari LGA and serves as the primary referral center for dog bite cases originating from health facilities within and outside the LGA. The veterinary facilities catering to animal health in Sabon Gari LGA include the state veterinary clinic, two private veterinary clinics, and Ahmadu Bello University Veterinary Teaching Hospital (ABUVTH), which acts as the major referral center. Previous reports documented regular dog bite cases at ABUVTH prior to this study, including referrals from outside the Zaria metropolis (36), while national surveillance data identified Kaduna State and the wider North Central region as having ongoing rabies transmission (26). The co-location of human and veterinary referral services therefore made Sabon Gari LGA a suitable setting for piloting IBCM and assessing its feasibility within the Nigerian health and veterinary systems.

### 2.2 IBCM development

Four consultation meetings were held in Sabon Gari LGA with the Directors of the State’s Ministries of Health and Agriculture, along with directors, nurses, and animal health workers from health facilities and veterinary hospitals/clinics within the LGA. These meetings were used to identify health and veterinary facilities operating within the LGA and those most frequented by dog bite victims, so as to designate IBCM focal health facilities and veterinary clinics. Community dialogues were held informally with the two district heads and eleven ward leaders along with available community aides, to secure their cooperation for piloting IBCM.

An IBCM research team consisting of one government veterinarian and a local animal health worker from Sabon Gari LGA was established. Their primary responsibilities included supervision, management, training, and retraining (wherever gaps exist) of the IBCM focal persons who are the health workers and veterinarians involved in the IBCM workflow. Staff at focal health facilities and veterinary clinics were selected as IBCM focal persons by the directors or heads of sections of the selected health facilities, veterinary clinics, and hospitals within Sabon Gari LGA.

**Figure 2:**
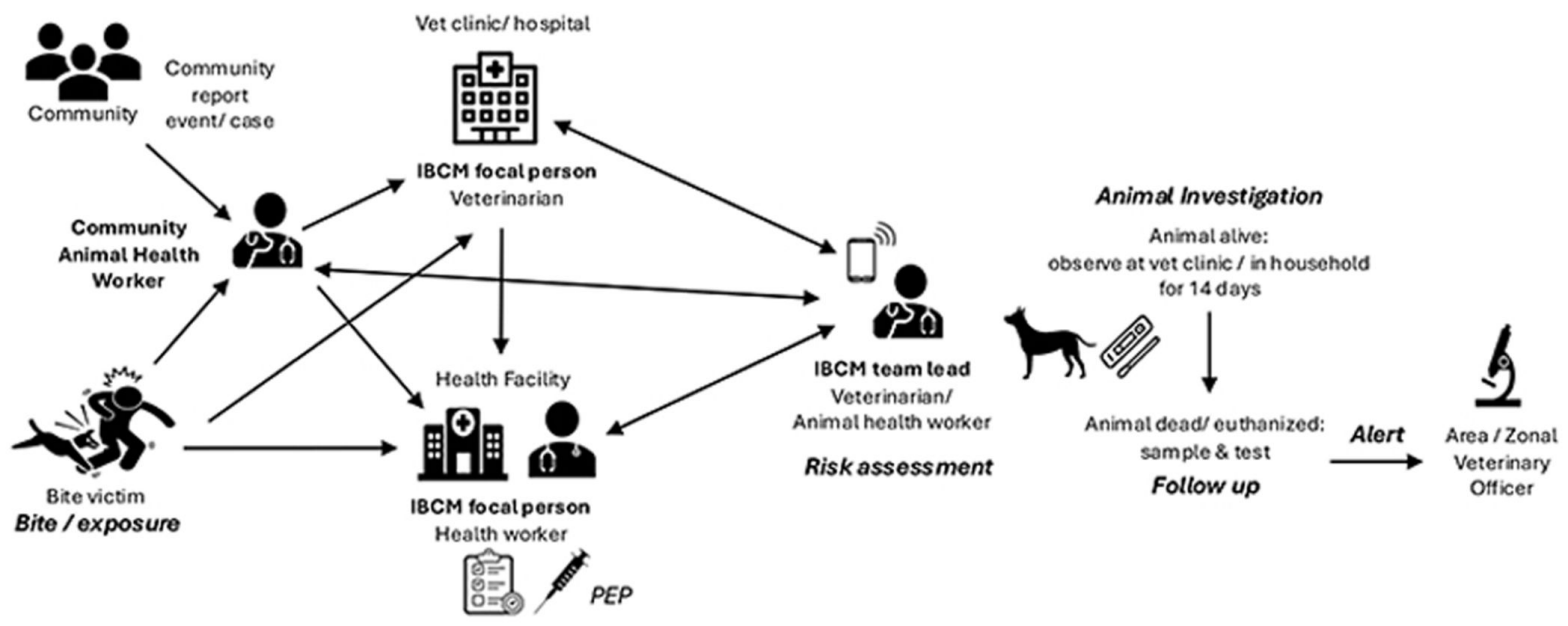
Workflow for integrated bite case management introduced in this pilot study. IBCM team members investigate biting animals following notifications of suspicious animals from health facilities, veterinary clinics and community members, and conduct rapid diagnostic testing on dead animals during follow ups. CAHWs are engaged to also refer bite patients to both veterinary clinics and health facilities with IBCM, while the IBCM team leader coordinated communications between health and veterinary personnel, facilitated by a WhatsApp group for all involved.

We then designed an IBCM protocol, comprising risk assessments of bite patients, animal investigations and communications between health and veterinary personnel (Figure 2). Bite patient risk assessments were designed to document bite victim details at the focal health facilities. IBCM focal persons at those health facilities were instructed to notify the IBCM team in the event of bite patient presentations. The IBCM veterinarian reviews the health facility register that captures wound categories, rabies risk factors, including details about the suspect animal and signs of rabies. This information helps to trace bite incidents and investigate the biting dog to determine whether PEP should continue or stop. The register also records exposure type, bite or scratch location and severity, animal species and vaccination status, behavior, geographic location, and circumstances of the incident.

The IBCM team was trained to conduct investigations in person and by phone and record data using KoBoToolbox via the KoBoCollect mobile app (37), based on risk assessment tools that were adapted from Tanzania (10) to the Nigerian context. Nurses were trained to complete patient registers and conduct risk assessments during visits. The veterinarian was trained to collect brain tissue using the straw method (38), to perform rapid testing with Bionote® kits (39, 40), and to package samples for laboratory transport. The rapid diagnostic tests (RDTs) were introduced to overcome delays in obtaining results from WOAH-approved dFAT used for rabies confirmation in Nigeria (41). Animal investigations captured bite incident details, assessed the animal’s health and vaccination status, and involved quarantine or sample collection for dead animals. Owners were advised to quarantine biting dogs for 14 days at home or in a veterinary kennel and report behavioral changes via phone. During quarantine, veterinarians monitored dogs through visits or calls, then completed investigations to determine the health or rabies status (Figure 2). RDTs were performed on-site for real-time decisions and coordination with health authorities.

The IBCM team were registered on the KoBoTool with the App downloaded to their phones. Preliminary IBCM field practice was conducted for the team to become familiar with the App. Practical sessions included simulation exercises to practice asking dog bite patients risk assessment questions, to investigate biting animals, to collect brain tissue samples, and to use the App. Subsequent meetings and IBCM training were held at Saint Jerome Veterinary Clinic, Sabon Gari LGA.

Animals that were investigated were classified according to WHO’s case definitions (42). Specifically, a suspect case was defined as an animal displaying any of the following clinical signs: aggression (unprovoked or abnormal), lethargy, paralysis, hypersalivation and abnormal vocalization; a probable case was defined as a suspect case with a reliable history of contact with a suspected, probable, or confirmed rabid animal, and/or that was killed, died or disappeared within 4–5 days of illness being observed. A confirmed case is one that tested positive for rabies by dFAT (42, 43), however we also considered RDT positives as confirmed cases. A non-case or healthy animal is one that remained healthy after a 14-day quarantine/observation period.

A suspected human case of rabies was defined as an acute neurological syndrome characterized by hyperactivity or paralytic syndromes that leads to coma and death within 7–10 days. Accompanying symptoms may include; aerophobia, hydrophobia, paresthesia, localized pain, dysphagia, localized weakness, nausea, or vomiting. A probable human rabies case is a suspected human case with a reliable history of contact with a suspected, probable or confirmed rabid animal (42). We considered people bitten by suspected, probable or confirmed rabid animals as a “high-risk bite” while a “low-risk bite” involves a bite by a healthy animal.

### 2.3 IBCM implementation

In April 2023, we commenced piloting IBCM, which extended to December, 2024. The IBCM research team maintained regular phone communication with, and visits to, focal health workers and their respective facilities, typically on a weekly basis, to ensure bite patients were documented and follow-up carried out appropriately.

### 2.4 Analysis

The human risk assessment and animal investigation data from the Kobotool dashboard were exported. Incidence was calculated from the records of probable rabid animals and the Sabon Gari LGA population projections for 2022 as denominators in the absence of recent census data (34).

Monthly time series of animal investigations classified into those determined to be healthy, probable, and RDT confirmed rabid animals were plotted based on the recorded date the victim was bitten. Time series of human risk classifications (low risk human bites, probable human exposure and probable human rabies deaths) were based on the dates patients were recorded as being bitten and developed symptoms respectively. Maps and time series were created using R (44).

The costs required for implementing IBCM, including fuel, phone credit, market collection, and transportation, were compiled and converted to USD based on the Central Bank of Nigeria exchange rate of $1 to 1,489 NGN as of April 2024 (45).

Investigations were conducted in a manner that ensured animal and owner safety, following WHO/WOAH guidelines. No financial compensation was routinely provided; however, in rare instances where dog heads had already been sold, reasonable reimbursement was offered to the butchers.

## 3 Results

### 3.1 IBCM development

The IBCM researcher’s consultations with the rabies lead in the Neglected Tropical Disease division of the Federal Ministry of Health and Social Welfare (FMoHSW) revealed that while in the past they supplied rabies post-exposure vaccines to ABU Teaching Hospital, before transfer to ABU Sickbay, this supply has ceased since 2015. Not all health facilities within the LGA were included in this pilot of IBCM. Facilities were selected purposively based on stakeholder consultations, which identified those most frequently visited by bite victims. Three main health facilities that were frequented by dog bite victims in Sabon Gari LGA were identified: The ABU UHS i.e. the sickbay, Major Ibrahim Bello Abdullahi (MIBA) State Hospital, and Primary Health Care (PHC) Kwangila, but none stocked PEP. Three private pharmacies located 5–11 km away were found to intermittently sell human rabies vaccines, typically stocking 5–20 vials each. The most common post-exposure vaccines sold were Rabipur® and Abhayrab® costing around $6 per vial. Rabies immunoglobulins (RIG), which are very expensive, were not available locally and are rare even in major cities. In Nigeria a complete PEP course involves five intramuscular injections (on days 0, 3, 7, 14, and 28) in the deltoid muscle and responsibility for paying for PEP lies with the dog owner or the bite victim.

The consultations mobilized human vaccine resources for the pilot. In preparation, the local NGO, War Against Rabies Foundation (WARF), spearheaded awareness campaigns and distributed educational materials in marketplaces, religious settings, and schools to enhance awareness and communication about rabies risks in the community. They also sought the cooperation of Community animal health workers (CAHWs). The Division of Neglected Tropical Diseases in the FMoHSW donated 50 vials of post-exposure vaccines in late June 2023.

The three health facilities identified above (ABU Sickbay, MIBA, and PHC Kwangila) were enrolled in the pilot (Figure 1B). These facilities were advised to request vaccines when bite patients presented, enabling them to be vaccinated for free. Three *ad-hoc* referrals subsequently prompted the inclusion of additional facilities: Alkausar Pharmacy, PHC Durumi (which was provided in June 2023 with 63 vials from the remaining vaccines given for pre-exposure vaccination of high-risk staff working with the State Ministry of Agriculture), and Barau Dikko Teaching Hospital (Figure 1B).

The hardcover hand-ruled notebooks used to capture basic patient information (visit and follow-up dates, names, reasons for visits, treatments administered) at health facilities and veterinary clinics were found to be insufficient for follow-up investigations. More detailed registers were therefore introduced (Appendix 1) that included information for animal investigations, such as bite victims’ names, dog owners’ phone numbers and addresses for follow-up consultations, quarantine locations and outcomes.

When rabies signs were observed and the animal was killed, died, or disappeared within 14 days following the bite, the IBCM team promptly reported the cases to the LGA Zonal Veterinary Officer who oversees animal health activities in the 8 LGAs of the northern Kaduna zone. Although this office is responsible for rapid outbreak investigation and response, including vaccination of exposed dogs, limited funding typically constrains these activities The IBCM pilot therefore supported the prompt execution of animal investigations. As illustrated in Figure 2, dog bite victims accessed care through multiple pathways, including referral by CAHWs to the IBCM veterinarian, or to human health facilities, and also by direct presentation to veterinary clinics, or human health facilities. Risk assessment in consultation with the IBCM team leader then triggered animal investigations, leading to quarantine or field sampling for RDT, followed by confirmatory testing by DFAT in the laboratory if reagents were available. Information from these investigations was then shared with the Area Veterinary Officer, for onward reporting to the zonal, state and national levels.

Key aspects of IBCM were refined to improve implementation during the pilot. For example, focal points at the IBCM health facilities did not upload questionnaires via the KoboTool App. Instead, they preferred to communicate through WhatsApp or phone calls. Consequently, we launched the Rabies Alert and Response Zaria (RARZ) WhatsApp platform in June 2023 to enhance communication, and provided 1.5 GB of phone data to six focal persons (Table 1). The costs of investigations varied, due to geographical factors and logistical differences in sample collection. For example, two investigations involved interception of dogs slaughtered for human consumption and their transport to the laboratory, while two dogs that died at the ABU VTH incurred no transportation cost. Total costs of IBCM over the 21 months implementation period amounted to $366 as shown in Table 1.

### 3.2 IBCM patient risk assessments and PEP

**Figure 3:**
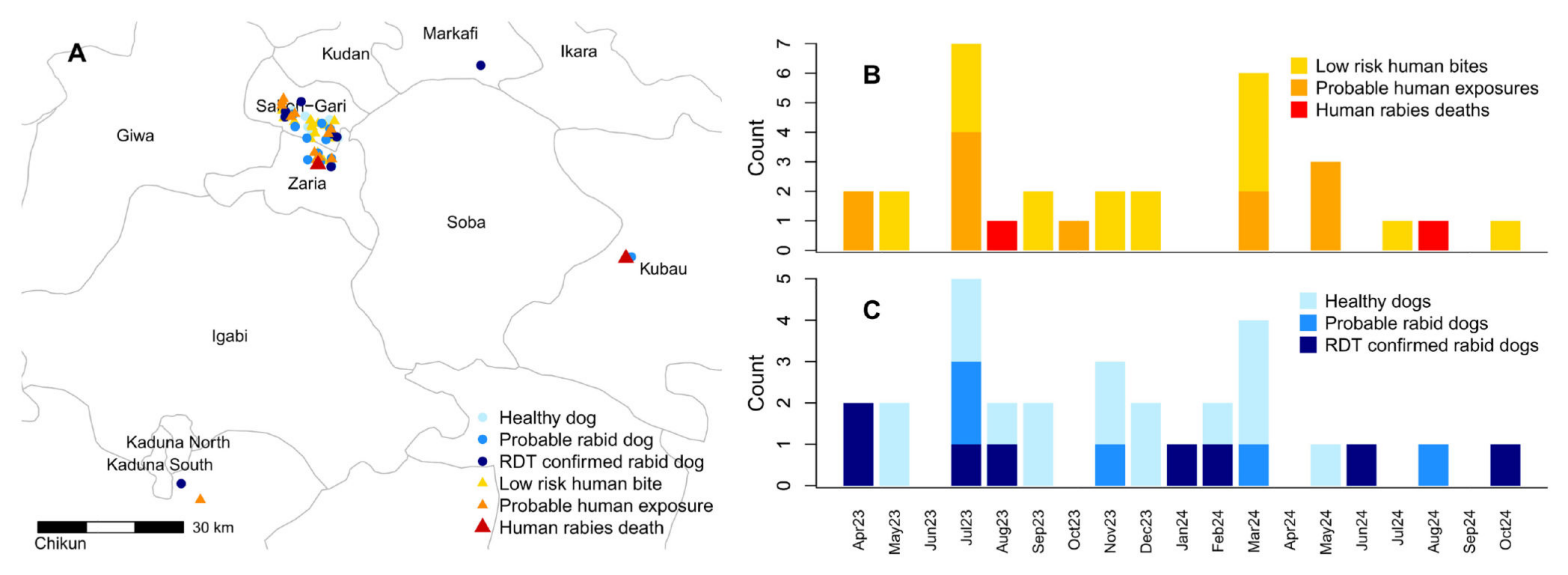
Human rabies deaths and probable rabies exposures and investigated dogs according to rabies status. **(A)** Locations of dogs investigated and bite patients who underwent risk assessments from April 2023 to December 2024. Time series showing **(B)** human rabies cases and high-risk and low-risk bites identified following completion of quarantine; and of **(C)** investigation results in terms of confirmed and probable rabid dogs and healthy dogs.

Between April 2023 and December 2024, risk assessments were performed for 28 dog bite victims (Table 2). An additional patient presented with signs of rabies and one human rabies death was also identified during investigation of a probable rabid dog. The ABU Sickbay received the most bite patients (33.3%, n=9, excluding the patient with rabies signs), followed by MIBA (23.3%, n=7). Kwangila and Durumi PHCs assessed five (16.7%) and three (10.0%) bite patients, respectively; three (13.3%) dog bite victims who presented at Alkausar Pharmacy were assessed, as was one patient who presented to Barau Dikko Teaching hospital (Figures 1, 3). These victims presented with bites from domestic dogs, primarily on the feet and legs (46.7%, n=14), and the arms and hands (40.0%, n=12), with two (6.7%) bitten on the head and neck, and another two (6.7%) with multiple bite locations (arms, hands, feet, and legs). At the health centers/pharmacies, all bite victims’ wounds were washed, and most received tetanus injections. Except for the two human rabies cases, all bite victims who presented to health facilities received their first post-exposure vaccination (93.3%, n=28). Subsequently 16 (53.3%) discontinued PEP as the dogs remained healthy after quarantine, while the remaining 12 (40.0%) completed PEP (Table 2).

During IBCM risk assessments 12 patients (41%) were classified as high-risk exposures (excluding the patient presenting with clinical rabies), while the remaining bite patients were classified as low-risk because the dog remained healthy (16, 53%) or could not be classified (1 unknown risk, 3%, Table 2). Five bite patients and the two human rabies cases had contact with probable rabid dogs (5 probable rabid dogs bit 7 people), while seven patients were bitten by confirmed rabid dogs (5 RDT-positive dogs bit 7 people). The two rabies deaths corresponded to a 13-year-old boy and a 50 year old man. One person bitten by the same dog that bit the young boy promptly sought care and received PEP, however, the boy could not afford PEP so did not seek care. Approximately five weeks later, the boy developed a fever, pain at the bite site, and later, aerophobia. He was treated for malaria but did not respond and subsequently died. The second older fatality was bitten in July 2024 while sleeping with his door open at 3 am, as is common among some rural communities during the hot season. Initially, he sought treatment from traditional healers. Later, influenced by the community ruler, he was taken to a veterinary clinic but was already showing clinical signs (hypersalivation, abnormal vocalization, and respiratory distress) and died shortly after.

The 28 bite patients comprised of residents from the LGAs of Sabon Gari (19), Zaria (8) and Kaduna South (1), while the two human rabies deaths were from Zaria and Kubai LGAs respectively (Figure 3). The annual incidence of bite patient presentations based on the projected population denominators from each LGA was 2.52 (95% confidence interval, 95%CI: 1.61-3.93), 0.76 (95%CI: 0.39-1.50) and 0.096 (95%CI: 0.005-0.54) per 100,000 persons/year for Sabon Gari, Zaria, and Kaduna South LGAs. Combining these figures gives an overall bite patient incidence of 0.98/100,000 people/year (95%CI: 0.68-1.42). The two deaths from Zaria and Kubau LGAs respectively, give an annual mortality rate of 0.11 rabies deaths per 100,000 people/ year (95%CI: 0.031-0.41). Since around 16% of persons bitten by rabid dogs develop rabies in the absence of PEP (46), we expect that around 10 other rabies exposures may have occurred who did not obtain PEP and who were not identified by our study (95% CI: 0-30). Based on the rabies exposures who received PEP (n=12) and those who did not (n = 2 deaths + 10 unobserved exposures), we estimate a rabies exposure incidence of 0.68 per 100,000 persons/year (95% CI 0.67-1.23) and a probability of rabies exposures receiving PEP of just 0.50 (95% CI: 0.27-0.86).

### 3.3 Animal investigations

**Figure 4:**
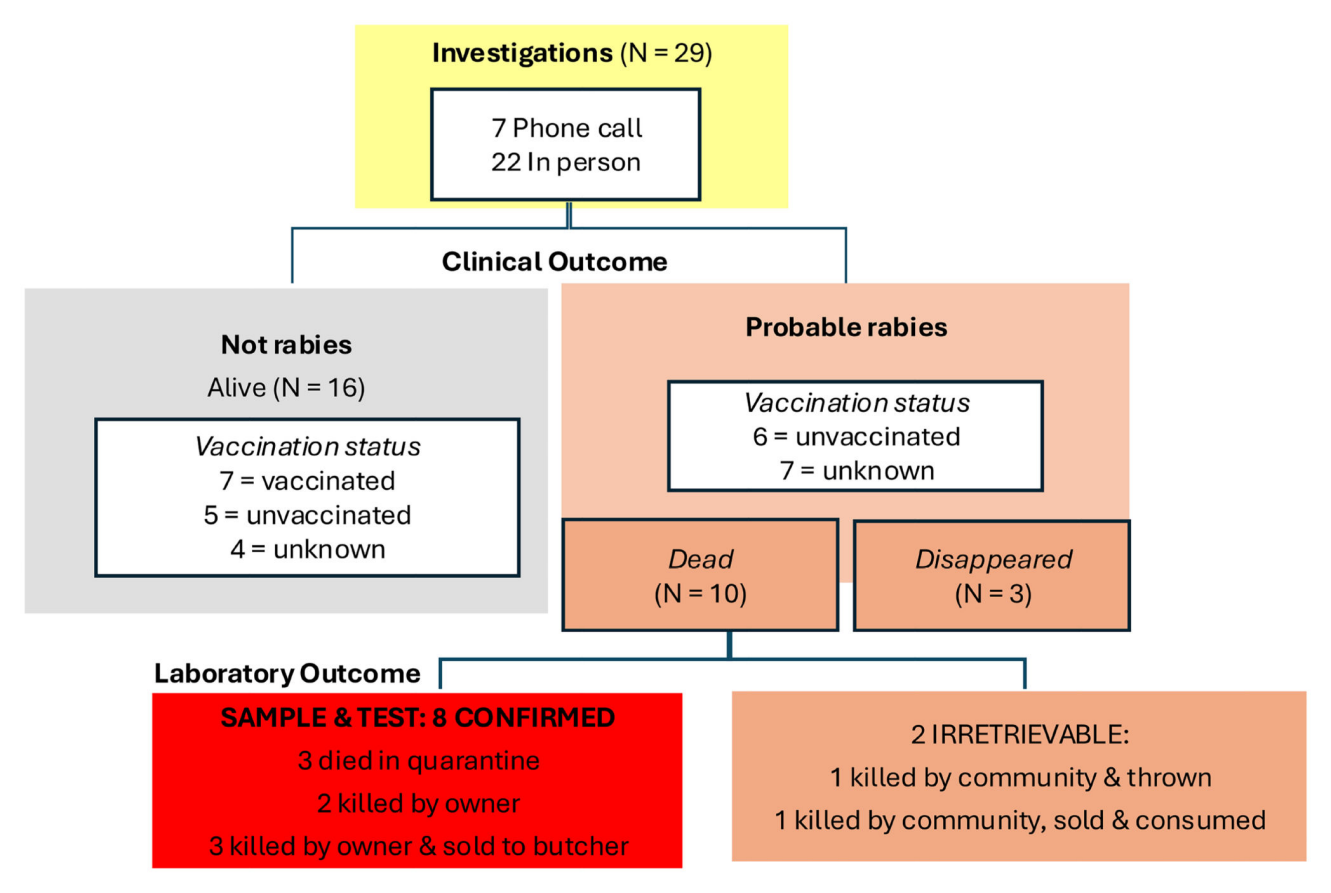
Outcomes of animal investigations in Sabon Gari, Zaria Metropolis, Nigeria (April 2023 - December 2024). Highlights the vaccination history, outcomes of dead and killed dogs and rapid diagnostic and fluorescent antibody tests.

The veterinary sector investigated 29 dogs during the IBCM pilot; 26 (93.1%) that bit people and three (6.9%) that were acting suspiciously (Figure 4). CAHWs referred the majority (37.9%, n=11) of the investigated dogs to veterinarians via phone calls, while a third came directly to veterinary hospitals/clinics (34%, n=10). Just over a quarter (28%, n=8) were identified when their bite victims went to health facilities before vets were engaged in the investigations. Of the 29 investigations, 23 were in-person (3 initiated by phone calls) and six were carried out over the phone only (Figure 4). The investigations identified five probable rabid dogs from clinical signs/history (17%) and eight rabid dogs (28%) were confirmed by RDT, of which three were identified following reports of clinical signs by CAHWs but were not reported to have bitten people. Sixteen of the investigated dogs (55%) remained alive and were categorized as healthy following the 14 day quarantine. The majority of these healthy dogs were unvaccinated (n=11, 37.9%).

Of the 13 probable rabid dogs three disappeared, and two were irretrievable for sample collection, after their carcasses were sold and discarded respectively. The remaining eight dogs all tested positive by RDT but only six were confirmed with DFAT, as testing reagents were not available for two samples. The IBCM team successfully intercepted two of three dogs that after being killed were sold to the dog market for human consumption, enabling testing (Table 1). The veterinarian attending to the dog that bit the patient who presented to Barau Dikko Teaching Hospital consulted with the IBCM team to assist in the investigation. The IBCM team was able to track and recover the dog sold by its owner to avoid the responsibility for the bite victim’s treatment. As this case was confirmed to be rabid, the assistance of the Director of Veterinary Services (DVS) and the Kaduna State Police, led to the dog owner paying for the victims’ PEP course.

## 4 Discussion

This implementation study piloting IBCM over a 21-month period revealed key insights into rabies management in Kaduna State, Nigeria. Prior to the pilot, PEP was not freely available for bite patients, prompting a donation of 50 vials by the Division of Neglected Tropical Diseases from the FMoHSW, to ensure that bite victims who subsequently presented received free vaccinations. During the pilot risk assessments were performed for 28 dog bite victims and two rabies deaths were identified; a young boy who did not have funds to seek care and an older man who visited a traditional healer but did not seek PEP. Of 29 dogs investigated, 26 caused biting incidents whereas 3 did not bite people but were identified after showing rabies signs. Eight dogs tested positive by RDT, 5 were considered probable and the remaining 16 had rabies ruled out after a 14-day quarantine. Of three rabid dogs sold for human consumption, two were intercepted and tested positive. These results indicate a relatively low incidence of bite patients, partly reflecting Kaduna’s predominantly Muslim population where dog ownership is uncommon (47). Yet, rabies remains prevalent and access and uptake of PEP was very poor.

Risky practices in Kaduna state prevail including the sale and consumption of rabid dogs (48–52), highlighting an urgent need for One Health approaches that strengthen rabies surveillance, control and prevention. The identification of confirmed and probable rabid dogs, including animals sold for human consumption, demonstrates ongoing transmission and exposes critical gaps in surveillance, risk awareness, and prevention. Together, our findings show that low bite presentation rates do not equate to low rabies risk and underscore the value of IBCM in revealing otherwise undetected transmission pathways. Strengthening access to PEP, improving community awareness, and enhancing coordination between human and animal health sectors are essential to reduce rabies mortality in Nigeria and support progress toward elimination goals.

### 4.1 Broader context

In rabies-endemic countries like Nigeria, the availability of PEP and a trained workforce to handle dog bite patients is crucial to rabies prevention. Through this IBCM pilot, inadequacies in Kaduna state’s health system were identified including a lack of PEP in health facilities, and poor knowledge about bite patient management among both private and public healthcare providers who required guidance from the IBCM team leader to recognize rabies risk. One of the human rabies deaths identified highlights the importance of removing financial barriers to ensure timely care seeking (7, 11, 13). Our estimate of very low levels of care seeking among rabies exposed bite victims further suggest an urgent need to improve awareness of the need for PEP among those at risk and to ensure PEP is accessible. Community sensitization activities by the War against Rabies Foundation have started to sensitize dog owners (17–19), but more serious investment by the national government is warranted.

We similarly observed very limited investment by the national government in rabies surveillance capacity, including for diagnosis. The funds and RDTs to support investigations as part of IBCM sparked enthusiasm among veterinarians. The immediate positive results fueled their motivation, enabling intersectoral feedback and appropriate PEP administration. All the samples they collected tested positive, in agreement with the bite history. This success instilled confidence in both field veterinarians and health workers, aligning with reports from other countries (10, 14, 53). The cost of the IBCM pilot was not large (Table 1), but exceeded current investment, reflecting the degree to which rabies is neglected (14).

Experience from this IBCM pilot highlights how surveillance activities must be mandated with training centrally coordinated by the federal government for IBCM to become routine. We found that the WhatsApp platform supported intersectoral communications about dog bite incidents. Although focal government healthcare workers were trained at the start of the pilot, they lacked confidence in identifying rabies signs and were reluctant to record data. Instead, the health workers engaged on the WhatsApp platform which enabled rapid information dissemination, allowing for real-time updates and collaborative management of animal rabies cases and bite patients. Examples of familiar communications platforms have been shown in other settings to enhance intersectoral communications for IBCM (10). However, these platforms alone are insufficient for reporting rabies cases and further investment in training for One Health practitioners as well as in health information systems is needed for effective rabies surveillance (54).

In Kaduna state, bite victims often seek veterinary support, after being referred from health facilities to veterinary hospitals. Veterinary professionals are supposed to assess the dog’s health to determine rabies risk, and provide crucial information for deciding on PEP. This process links the health and veterinary sectors, but places a burden on the patient, which could unnecessarily delay initiation of PEP, suggesting room for improvement. Dog owners’ vigilance in containing and reporting dogs with suggestive rabies signs can prevent further exposure. The use of quarantine was found to be valuable means of ruling out rabies. In this IBCM pilot, two dog owners promptly restrained and alerted veterinarians when their dogs exhibited abnormal signs. CAHWs were also proactive in contacting veterinarians with information about dogs showing signs suspicious for rabies. This positive engagement among the community is a valuable starting point for improving rabies control practices. Their participation in reporting suspicious cases and dog bites could be capitalized on to strengthen surveillance (53). Similar contributions have been documented in Cameroon, Chad and Mali, where CAHWs have played key roles in detecting and reporting zoonoses (55).

The improper disposal of dogs and consumption of rabid dogs poses serious public health risks (49, 50). Unfortunately, poverty-driven behavior sometimes leads dog owners or community members to sell potentially infectious biting dogs (50). In our study, three dogs were sold under such circumstances. These practices create multiple exposure points for rabies transmission to humans including traders, butchers, consumers, and bite victims, thereby sustaining rabies as a public health threat. Such activities facilitates human exposure during handling, slaughter, and meat preparation, likely explaining the high prevalence of rabies antigens detected in slaughtered trade dogs across Nigeria (48, 49). Dog trading hinders the confirmation of suspicious dogs and subsequent timely bite victim management, affecting outbreak response. Moreover, the movement of unvaccinated dogs for meat amplifies rabies transmission risk to traders, butchers, and consumers, creating a silent pathway for disease spread (51). Similar challenges exist in other rabies-endemic areas in Asia and Africa where dog consumption is practiced (56, 57). However, countries like Cambodia and China are actively working to end the pet trade (58). Our study highlighted the importance of engaging dog butchers, hunters and traditional healers as key stakeholders, who should be involved in rabies surveillance and its control.

Our Kaduna findings align with some experiences from IBCM implementation elsewhere. For example, practitioners in East Africa similarly embraced RDTs with enthusiasm which improved case confirmation (10, 53, 59), while use of CAHWs to support investigations also extended geographic reach as seen in Vietnam and Kenya (53, 60). However, we observed much lower levels of care seeking than in Tanzania (10), Haiti (61), Vietnam (60), and the Philippines (11), reflecting not only the sociocultural heterogeneity of religious mix, dog−keeping patterns, and the dog−meat trade, but also the very limited PEP access in Kaduna state. Health workers in Sabon Gari were also slow to adopt digital systems; programs that succeeded matched platforms to user habits and workflows for example, the national e−IBCM and REACT in Haiti (62) and the use of Facebook messenger in the Philippines (11). At the patient level, affordability blocks many patients from initiating and completing PEP (7, 63). Our experience in Nigeria is consistent with many other low-income settings where vaccine stock−outs, travel costs, and user charges delay and reduce PEP uptake (10, 63). In Haiti, funders who withdrew support for dog vaccination forced greater reliance on costly human PEP and increased mortality risk, illustrating how upstream financing gaps amplify patient−level barriers (64). These findings support waiving or subsidizing user fees, and leveraging Gavi support to stabilize supply and reduce patient costs (2, 7).

### 4.2 Strengths and limitations

The IBCM pilot led to improved data collection and active community involvement in rabies surveillance within the LGA. However, we encountered numerous challenges in implementing IBCM. We selected only a subset of health facilities for the pilot and none had PEP in stock prior to the study, while referrals from facilities outside of Sabon Gari LGA highlighted the difficulties that bite victims face in obtaining PEP. Our experience is that when PEP is not available bite victims are less likely to present to health facilities, often turning to traditional healers and those who develop rabies go unrecorded (as witnessed for the younger rabies victim). We also did not involve all veterinarians operating within the study area. Consequently, some bite patients and suspect rabies cases managed or recorded in non-enrolled facilities may have been missed, contributing to under-ascertainment.

Moreover, among the bite victims who presented to the enrolled IBCM facilities, several attended from other LGAs. Our estimates of incidence may therefore not reflect the true disease burden affected by bias and with denominator populations that were hard to define. Additionally, data was incomplete for certain variables, including untraceable biting animals that could not be observed or tested, and missing vaccination records for dogs. These gaps may have affected the accuracy of rabies classification. Moreover, case definitions used in Nigeria typically exclude probable cases, categorizing them only as suspect, confirmed rabies, or healthy animals and so do not align with WHO guidance (41). Official rabies records therefore are even more likely to underestimate rabies incidence.

Lack of resources and scope in expanding the study to include qualitative data collection on the IBCM team (including focal persons) limited our understanding around understanding the experiences of doing IBCM including acceptability, appropriateness and barriers and enablers of delivering IBCM. In addition, we did not apply an established implementation research framework, to structure the documentation and interpretation of our findings (65). We also did not evaluate training, which limits our ability to distinguish the effects of IBCM implementation from underlying levels of workforce preparedness. Finally, although the small geographic scope of the pilot (focused in a single LGA but necessarily covering incidents in a small number of nearby LGAs). Finally, although the small geographic scope of the pilot (focused in a single LGA but necessarily covering incidents in a small number of nearby LGAs) limits its generalizability, we anticipate that useful lessons can still be taken forward for the future development of IBCM in Nigerian contexts.

### 4.3 Conclusions and recommendations

Nigeria, like many other dog rabies-endemic countries, faces major challenges in rabies control and prevention. This study is among the first documented efforts to operationalize IBCM in Nigeria, addressing a critical gap and offering lessons for future interventions. A One Health approach using IBCM presents opportunities to address these issues, aligning with Nigeria’s national strategic plan to eliminate rabies by 2030. However, further development work is needed for effective implementation of IBCM in different Nigerian contexts. The IBCM pilot highlighted critical gaps in rabies control and prevention in Kaduna, most notably low levels of seeking PEP among rabid dog bite victims, low vaccination coverage in domestic dogs and high-risk practices within the dog-meat trade.

To address these poor practices, several policy-relevant recommended actions can be made based on the IBCM pilot. Engaging and supporting CAHWs and improving intersectoral communication and reporting mechanisms can enhance surveillance. Increasing dog vaccination coverage is crucial to reducing rabies risk and preventing transmission. Incorporate RDTs into national algorithms for field triage and immediate decision-making is required, together with standardized pathways (and updated case definitions) for diagnosis. Public education campaigns are essential to raise awareness about the importance of timely PEP, and to dispel myths surrounding traditional treatments for rabies.

To ensure that control and prevention efforts translate into measurable impact, they should be coupled with integration of IBCM into national surveillance platforms, enabling real-time, cross-sector alerts, harmonized data, and joint outbreak investigations supported by routine data sharing and feedback loops at LGA and state levels. There is a need for functional joint human and animal health coordination mechanisms with defined budgets, accountability indicators, and periodic reviews to track IBCM performance and accelerate progress toward elimination. Finally, Nigeria is eligible for human rabies vaccine support through Gavi, the Vaccine Alliance, and should therefore urgently apply so it can address gaps in PEP availability, especially in underserved communities. Overall, our findings underscore the importance of community involvement, enhanced surveillance, and public education in mitigating rabies risk. By addressing these gaps, it is possible to improve public health outcomes and reduce the incidence of rabies deaths.

## Figures and Tables

**Table 1:** Costs incurred during IBCM pilot in Sabon Gari LGA, Nigeria.

Item	Quantity	Cost/item ($)	Total ($)
In-person investigations at LGAs (n=23)			
i. Sabon Gari	16	6	96
ii. Zaria	4	12	48
iii. Others LGAs	3	15	45
Interception of sold dogs at LGAs (n=2)			
i. Kaduna South (Barau Dikko)	1	19	19
ii. Sabon Gari (Muchia)	1	13	13
Sample Transportation from LGAs (n=6)			
i. Kaduna South (Barau Dikko)	1	10	10
ii. Sabon Gari (Muchia)	2	6	12
iii. Sabon Gari (Bassawa)	1	4	4
iv. Kubau (Achau)	1	10	10
v. Zaria	1	8	8
Phone credit			
	126 (6 × 21 months)	0.805	101.43
Total			366.43

The pilot took place from April 2023 to December 2024.

**Table 2:** Outcomes of IBCM in terms of human rabies cases, risk assessments, PEP completion and biting animal investigations during the IBCM pilot.

Category	Status	N	Comment
Human rabies cases	Showed signs (presented to facility)	2(1)	The 2nd victim was only identified in the community
	Initiated PEP	0/2	Neither started PEP
Bite patient risk assessments	High risk (unknown risk)	12 (1)	Excluding the rabies patient that did not present to a facility
	Low risk	16	
PEP status	High risk completed PEP	12	
	Low risk completed/ initiated	0/16	All discontinued PEP when rabies ruled out after dogs survived quarantine
Biting animal investigations*	Observable & survived quarantine (+ vaccinated)	16 (7)	
	Observable & died in quarantine	3	
	Unobservable (Dead, killed or disappeared)	5	
	Sold/consumed	3	
	Confirmed rabid dogs*	5	Caused 7 exposures; *excludes 3 confirmed rabid dogs that did not cause exposures

Note that the details of the animals investigated that did not have contact with people are not included.

## Data Availability

The original contributions presented in the study are included in the article/supplementary material. Further inquiries can be directed to the corresponding author. A de-identified version of the dataset is available from the Github repository: https://github.com/boydorr/ZariaIBCM.
